# Successful programmatic approaches to facilitating IUD uptake: CARE’s experience in DRC

**DOI:** 10.1186/s12905-019-0793-3

**Published:** 2019-07-24

**Authors:** Sarah Castle, Heidi Schroffel, Jean Jose Nzau Mvuezolo, Bavon Mupenda, Justin Mumbere, Rachel Shapiro

**Affiliations:** 1 Independent Consultant Sarah Castle Consultancy Ltd, 37 Warren St, Fitzrovia, London, W1T 6AD UK; 2 CARE International USA, Atlanta, USA; 3 Ecole de Santé Publique, Université de Kinshasa RDC, Kinshasa, Congo; 4 CARE International DRC, Kinshasa, Congo

**Keywords:** IUD uptake, FP uptake, Determinants of FP use, Determinants of IUD use, Programmatic factors, Programmatic approaches, Low-resource setting, Method mix

## Abstract

**Background:**

Achieving the unfinished agenda towards sexual and reproductive health and rights requires overcoming remaining barriers to contraceptive uptake, which can be method-specific. Women’s uptake of the IUD is poor across sub-Saharan Africa. The objective of this paper is to identify the reasons for comparatively high IUD use observed in a CARE project in DRC, together with the programmatic characteristics which facilitated uptake.

**Methods:**

Qualitative data were collected in 2015 as part of a reproductive health project in the DRC. Using purposive sampling, 15 focus group discussions took place with IUD users, users of other methods and non-users of modern contraception as well as their male partners. Eighteen in-depth interviews were conducted with health providers, project staff, community health workers and local stakeholders to capture a range of experiences. Data were analyzed using content theory approach and contextualized through a review of routine monitoring data.

**Results:**

In an area with practically no previous IUD use, 38,662 new FP clients were served during the first 5 years of the project and 82% (31,569) chose long-acting or permanent methods. Over 10,000 clients chose an IUD, representing 30% of the total FP clients. Key informants expressed mainly positive views about the IUD and quality of service. Concerns related to method insertion, which some perceived as too intimate or shameful. Findings indicate that this uptake reflects effective supply chains, good provider training and supervision and multiple communication strategies including those which target men. Community engagement was enhanced by local stakeholders’ participation in sensitization and quality assurance as well in analysis of data for decision-making.

**Conclusions:**

The findings of the paper showed that by involving local stakeholders in addressing structural and socio-cultural barriers to women’s free access to FP, programs can positively influence quality of service and method mix as well as knowledge and attitudes surrounding FP use and thus improve the uptake of FP in general and IUDs in particular, even in conflict-affected settings. A Theory of Change for enhancing IUD provision within family planning programs is suggested.

## Background

Achieving the unfinished agenda towards sexual and reproductive health and rights requires overcoming remaining barriers to contraceptive uptake, which can be method-specific. Women in Africa have an unmet family planning need of 23%, the highest of any region in the world. Over 82% of family planning users in Sub-Saharan Africa rely on short-acting methods [1]. Real progress towards reducing unmet need cannot be achieved without a comprehensive method mix, which includes long-active reversible and permanent methods (LARC), given that some of the disadvantages of short-acting methods, such as “low adherence, high discontinuation and low switching to alternative methods after discontinuation” significantly contribute to unmet need [2, 3]. Use of IUDs in particular remains low. The percentage of modern method users using IUDs ranged from 0% of the 2014 method share in the Central African Republic, Chad, Congo-Brazzaville, Sierra Leone, and South Sudan to highs of 12% in Burundi and 37% in Guinea-Bissau [4]. Specifically, in the Democratic Republic of Congo (DRC), only 0.5% of urban women and no rural women were using the IUD at the time [5].

While there are conflicting findings over whether IUD use is more strongly affected by the attitudes of providers or clients [6], both sociocultural and health systems-level barriers contribute to a weak adoption of the method in Africa. Across the continent, reticence to use IUDs as well as discontinuation is associated with a fear of side-effects (including dysmenorrhea) and also with misinformation including the widespread belief that the method may cause long-term sterility [7, 8]. This is an important consideration for women whose status in their marital families and in society is often contingent on their proven fertility [9, 10]. Such rumors also lead to negative perceptions of the IUD within the community and by men. Other rumors are prevalent across the continent including the widespread belief that the IUD will get ‘lost’ in the woman’s body or appear embedded in an infant’s head or body at birth [11, 12]. Structural barriers include cost, fear of the pelvic examination necessary for insertion and an associated preference for female providers [13], a lack of provider training to insert or remove IUDs [6, 13], and provider misconceptions about eligibility, such as the belief that the method is not suitable for nulliparous women or HIV positive women [6, 14]. Providers might also favor other methods that require less time to provide. In addition, a lack of outreach in rural areas reduces access for many women given the need for trained providers and hygienic settings for insertion [15, 16].

In order to reduce unmet need, method-specific barriers to highly effective contraceptives like the IUD need to be addressed with increased effort from both the supply and demand side. At the same time, policy makers and program implementers need to be careful to ensure that these efforts expand, not narrow, women’s options by over-promoting any one contraceptive method. With this in mind, and in the context of intensifying global efforts to increase LARC uptake generally and IUD use specifically, we aim to strengthen the existing knowledge of effective FP program components and factors contributing to women’s choice of IUDs in low-resource settings.

This paper draws from a CARE initiative in three health zones of the DRC, which saw a significant increase in IUD use after addressing supply and demand barriers to access to FP in general and IUDs in particular. Among new FP users, nearly 40% chose an IUD at the end of the first 5 years of the project, compared to 0% prior to project implementation and 10% during the first 2 years of the program. We use qualitative data collected through this project to examine the reasons for this comparatively high IUD use observed in the project’s routinely collected quantitative data, together with the programmatic characteristics which facilitated uptake.

We hypothesized that the following demand and supply elements influenced IUD uptake in the project areas: perceived benefits and disadvantages of the method, the endorsement of contraception by religious and cultural gatekeepers, stigma around pelvic insertion of the IUD, and providers’ contraceptive counseling influence and confidence with IUD insertion. We further hypothesized that by addressing these issues through supply and data management, clinical training and engagement to address misconceptions, bias and harmful gender attitudes among providers and communities, we would positively influence availability of a wide range of FP methods, which in turn would lead to increased IUD uptake. We then sought to understand to what extent these results reflected underlying unmet demand of IUDs and how the findings might help providers, programmers and policy makers improve unbiased access to high-quality IUD service for women by proposing a theory of change.

### Study context

In 2013, the population of the DRC was estimated to be 80 million [17]. The country is amongst the poorest nations of the world [1] and also suffers from prolonged conflict, with more than 2.3 million displaced persons and refugees within DRC and 323,000 DRC nationals living in refugee camps outside the country [18]. Given the myriad social and structural challenges faced by women living in the DRC, FP use, and in particular access to LARCs, remains relatively low while the total fertility rate remains high, at 6.6 nationally with a median age at first birth of 19.9 years [19]. Access to FP services is limited, especially in rural areas. Of the 516 health zones in the country, less than half (46%) have FP services [17]. Unmet need for FP among women of reproductive age has been estimated at 28% [19]. The 2013–2014 DHS indicated that among all women surveyed in DRC, the preferred method of choice comprised traditional methods, used by 11% of respondents. Although 15% of urban women (compared with 5% of rural women) used a modern method, these were mainly condoms (6%) and injectables (2%). While around one quarter (24%) of women surveyed had heard of the IUD, only 0.5% of urban women and no rural women were using this method at the time of the 2013–14 DHS [5]. Evidence suggests that women often progress ‘step-wise’ to use LARCs, starting with a short-acting method and then switching to a long-acting method if they are satisfied with it [8]. IUD use in the DRC is therefore challenging given the small numbers of women using any modern method at all.

While IUD use remains low in the DRC, focusing on quality service provision holds promise for meeting unmet FP need. A review of Marie Stopes International’s programs to increase the uptake of implants in sub-Saharan Africa provides a number of recommendations for implant uptake. These include a focus on those with unmet need, raising awareness, diffusing messages using multiple communication channels, delivering high quality services and generating and meeting demand among women in different geographical areas and social strata through strategic partnerships between stakeholders, including clients [20]. Hancock et al. emphasize the need to renew focus on service quality with a view to enhancing provision and uptake [21]. They cite the framework developed by Bruce, which necessitates a choice of methods, accurate information given to users, the technical competence of providers, respectful interpersonal relations, adequate follow-up and continuity mechanisms and an appropriate constellation of services [22]. However, there is a gap in knowledge about if and how communities themselves perceive and value the FP programs which target them, particularly those which offer LARCs at the community level.

An additional review of factors contributing to successful FP programs noted that they also require a strong government vision with leadership and commitment to FP [23]. For example, in Ethiopia, Desta, Brahmi and Alemayehu note that IUD use has increased considerably in large part due to government support for community outreach comprising effective training and supervision for insertions by community-based Health Extension Workers [24]. Tilahum et al. reported that the Ethiopia Intrauterine Contraceptive Device Scale-up Initiative (2011–2013), which was extended through 2017, increased the percentage of modern contraceptive users receiving an IUD from 0.4 to 5.7% by 2014 [13]. Together with adequate financial and human resources, this can create an ‘enabling environment’ for effective local service delivery.

### The SAFPAC project in DRC

Under the *Supporting Access to FP and Post-Abortion Care* (SAFPAC) initiative, in 2011 CARE established a project to expand the provision of selected reproductive health services in the Kasongo, Lubero and Kayna health zones in the provinces of Maniema and North-Kivu in DRC. In December 2015, program activities were discontinued in Kasongo health zone but continued in Kayna and Lubero and in additional zones of Goma and Butembo. Further research is necessary to establish whether FP activities continued at the same scale in Kasongo after CARE withdrew.

The project placed emphasis on addressing barriers that had been identified specifically to IUD use, such as provider misconceptions and discomfort with method provision, and rumors that existed in the community. In the three initial SAFPAC health zones, provider training included FP counseling, as well as technical training in the insertion and removal of implants and IUDs, post-partum counseling and post-abortion care. Together, CARE and the Ministry of Health convened meetings for health care providers as well as for community representatives. For logistical and financial reasons, not all providers received all training modules. Between 2011 and 2015, a total of 54 providers (8 physicians, 22 nurses and 24 auxiliary nurses) in 21 clinics throughout the three health zones were trained in IUD insertion. Nearly half of the trained providers (46%) were female, and the majority (62%) of all of those trained were in public health services whilst 11% were in the private sector and 28% in faith-based services. Supervisory visits were carried out by CARE or local Ministry of Health staff or both who conducted skills assessments and gave follow-up support. All methods were made available free of charge. While CARE supplied all facilities with contraceptives, commodities and equipment, it worked closely with the Ministry of Health to increase sustainability through capacity building, joint decision making and knowledge dissemination.

SAFPAC also adopted a holistic approach to working with communities in order to address social and gender norms that inhibit women’s access to services. Community Health Workers (‘relais communautaires’) and community facilitators (often affiliated to women’s groups known as Misadi) were trained in Social and Behavior Change Communication techniques. These groups of women played a critical role in increasing the awareness and acceptance of FP services. They emphasized healthful group behaviors and communication while also addressing the social context, systems, and processes that underpin health seeking behavior. Their sessions were enhanced by the creation of Quality Fora. These comprised local meetings with providers, stakeholders and community members which served to monitor service quality and to hold health workers accountable. Printed materials and radio broadcasts accompanied the sensitization sessions and fora meetings.

In the SAFPAC zones of intervention, providers routinely collected data about service delivery and client characteristics. Verification of data quality included data validation and triangulation between three data sources (client cards, registers and monthly reports). The data were collated from these sources on paper and transferred into Excel spreadsheet summaries (which have data validation mechanisms in place) and rolled up to the global project team. The project placed a strong emphasis on data for decision-making in collaboration with community stakeholders and implemented program adjustments after trends or gaps in coverage had been identified.

**Fig. 1:**
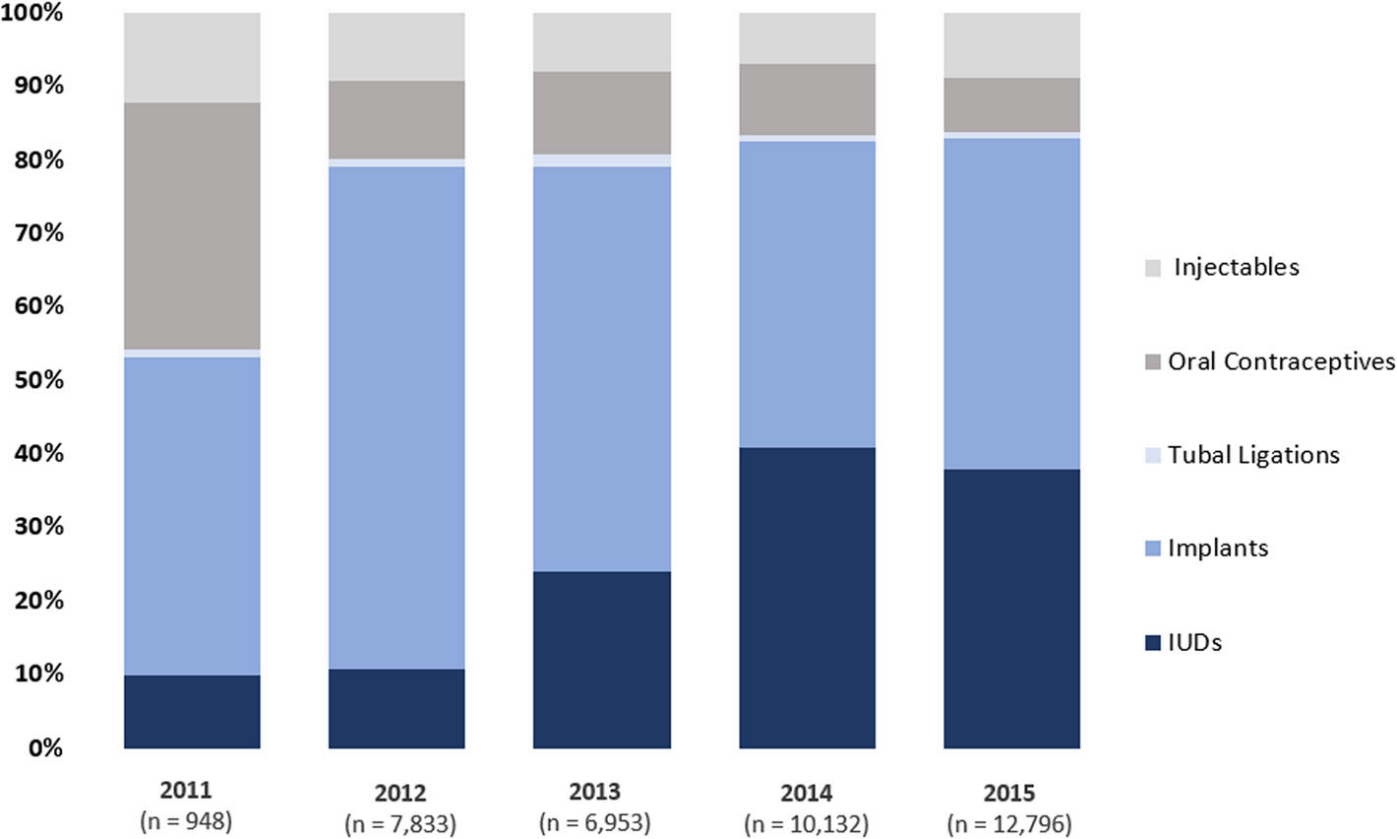
Distribution of Contraceptive Method among New Family Planning Users. (NB 2011 Q2 Q3 Q4 only, 2015 Q1 Q2 Q3 only. Number of vasectomies too small to show)

The analysis of this routine data demonstrates high voluntary LARC uptake in the DRC through the SAFPAC project. Figure 1 indicates that the IUD is one of the most preferred modern contraceptive methods (second only to implants) in the SAFPAC project intervention areas in DRC. Out of 38,662 new clients who benefited from a modern FP method in the 21 supported clinics during the first 5 years of the project, 82% (31,569) opted for long-acting or permanent methods (IUD, implant, tubal ligation, vasectomy). Over 10,000 clients chose an IUD, which represents 30% of the total FP clients and 37% of all users of LARC methods.

## Methods

Qualitative data were collected via a cross-sectional, descriptive study methodology and selection of participants focused on providing rich information about the research question from various perspectives. Table 1 presents the distribution of in-depth interviews and focus group discussions that were carried out with key informants comprising IUD users, users of other methods and non-users of modern contraception together with the partners of IUD and other modern method users. In addition, providers, CARE staff, community health workers and facilitators and local stakeholders were interviewed.

**Table 1:** Distribution of in-depth interviews and Focus Group Discussions

Type of Participants	Number per Health Zone		
	Kasongo	Kayna	Lubero
In-depth Interviews with Key Informants			
Staff of CARE International DRC	2	2	2
Community Facilitator (Partners of CARE)	2	2	2
Health Care Providers from Health Zones	2	2	2
Total In-depth Interviews	**6**	**6**	**6**
Focus Group Discussions			
IUD users	1	1	1
IUD users’ sexual partners	1	1	1
Non-Users of Contraception	1	1	1
Users of Other Methods of Contraception	1	1	1
Sexual Partners of Other Methods of Contraceptive Methods	1	1	1
Total Focus Group Discussions	**5**	**5**	**5**

Among all project-supported health zones in Maniema and North Kivu, four sites or health areas, defined as health facilities with surrounding catchment areas, were purposively selected to be included in the sample under considerations of accessibility.

Key informants for in-depth interviews were selected based on their involvement and knowledge of the program in order to represent a range of experience among implementing stakeholders. Participants for focus group discussions were selected among women of reproductive age, both married and unmarried, who lived in one of the four selected health areas. They were recruited purposively using community contacts.

Most respondents were under 35 years of age and most had primary level education. The majority were Catholic although a few respondents, mostly from Kasongo, identified as Muslim. Twenty-four interviewers (8 interviewers per health zone) were selected among data collectors previously trained by CARE during a three-day workshop on qualitative interviewing and facilitation techniques and tools as well as informed consent. Interviewers practiced using the guides through role play and carried out pilot interviews after which the guides were refined. Interviews and focus group discussions were recorded using digital recorders in order to facilitate transcription. Prior to the study, the research protocol was reviewed and approved by the Ethics Committee of the Kinshasa School of Public Health. Verbal informed consent was obtained from each participant before the interview.

Interview and focus group discussion guides were developed in French and translated into Kiswahili by professional translators. Both research activities were conducted in Swahili or French as appropriate and depending on the environment and then translated directly into English by a qualified translator. Due to resource limitations, no back-translations were carried out; however, all translated transcripts were checked by supervisors fluent in the relevant languages for quality control and accuracy.

### Data analysis

All transcriptions from the in-depth interviews with key informants and from focus group discussions were typed into MS Word 2007 and the files then exported into plain text format to Atlas.ti 5.2 software. The data then underwent a preliminary qualitative analysis comprising a systematic classification involving coding and identifying themes or patterns [25]. Analysis started during data collection so that themes which deserved further exploration were recorded and incorporated into the interview guide for subsequent field work. After data analysis was completed, additional analysis of the transcriptions was carried out by a second researcher in order to increase the objectivity of the interpretation and findings. The second set of analyses used The Ethnograph Software (Version 6). This involved formatting the original transcripts to make them compatible with the program including automatically numbering the lines of text. Parent codes and ‘sub-codes’ were then developed and applied to different segments depending on the content. For example, a subcode could be ‘DISADVANIUD (disadvantages of the IUD) or DISADVANIMP (disadvantages of the implant) and the ‘Parent code ‘DISASDVANALL (disadvantages of all methods). The codes were then sorted and cross-referenced and the segments analyzed for content to isolate norms and behaviors as well as their context.

The development of coding categories was guided by a ‘content theory’ approach [26]. Content analysis involves coding segments of text which correspond to the different topics that appear in the transcriptions. Subsequently, coded segments on the same topic are classified and compared across all interviews and discussions, with the aim of developing theoretical constructs from the data. The content theory approach looked at reasons for preferred method choice (particularly around IUD as preferred method); client (and partner) satisfaction with the chosen method (especially with IUD use); client satisfaction with service provision especially around IUD insertion and removal; provider perspectives on training and service delivery atogether with cultural and religious factors around FP (and specifically IUD) uptake.

## Results

Evidence from testimonies from focus group discussions and in-depth interviews is presented below in relation to IUD uptake and provision from a range of perspectives, focusing on both demand and supply factors. The discussion and quotations shed light on the individual (provider and client) factors as well as community-level factors which are likely to be associated with relatively high levels of IUD use.

### Findings on demand factors related to IUD uptake

#### Demand creation

The project used engagement strategies targeting women, men and community stakeholders, with the goal of overcoming socio-cultural barriers, addressing lack of accurate information, and ultimately generating demand for FP, including IUDs. Community facilitators and women’s groups reached out to women through radio and in the privacy of their homes, in savings or religious groups as well as during antenatal consultations. Both providers and community facilitators are part of the communities they live and work in and can sometimes perpetuate negative perceptions about IUD use. At the same time, they play an important role in breaking down barriers to FP access. In the case of this study, both community facilitators and providers affirmed their conviction of the benefit of women’s unrestricted access to a broad range of methods. They reported placing particular emphasis on overcoming the potential shame associated with the pelvic examination and insertion, an approach they perceived as effective in increasing IUD use.

Each time we give out messages during antenatal care there are always women who sign up for an IUD during the following days. [ … ] We need to talk about the side-effects so that people understand them and at the end we need to get rid of notions of shame about nudity and to make this taboo go away*.*

IDI, Female provider, 40-50 years old, Kasongo.

They also reported increased awareness and acceptance of FP and IUDs among both women and men in the community, a finding that also emerged among focus group participants, including men, who cited a perceived lack of side-effects as one of the reasons to support IUD use.

As far as I’m concerned, I’d recommend the IUD. There are no side-effects*.*

Focus group discussion, partners of IUD users, married man, 50-60 years old, primary education, Lubero.

Nevertheless, while they valued feeling informed and acknowledged the benefit of FP use, some men were not keen on women autonomously seeking out an IUD or another method of FP and felt that their permission was needed. They emphasized that messaging should encourage women to bring their husbands along to the consultations, in some cases stating that they would seek legal action against providers if their consent was not obtained.

I don’t like it if a woman uses family planning without the consent of her husband. You need to carry out the sensitization so that men have the same level of information as women. Regarding your health providers, they need to make sure that women don’t come alone to the health center to get an IUD or another method*.*

Focus group discussion, partners of users of other methods, married man, 30-40 years old, secondary education, Lubero.

The most common reason cited for this opposition to women obtaining FP without their consent - particularly IUDs - was jealousy, followed by perception of undermined authority. A close, trusting relationship between the provider and a female client – a necessary component of successful rights-based FP programs – was met with suspicion from some men, who perceived such a relationship as too intimate. They also felt ill at ease at the thought of their partner being nude in front of a potentially male provider. Many female focus group participants emphasized the need for male engagement in gender-transformative activities, beyond mere transmission of information. They felt this would help them avoid arguments or the necessity for secret FP use in the face of male disapproval. Their aversion to secrecy suggests that women were not only seeking autonomy in their reproductive choices, but also desired increased involvement from their partners in decision-making around FP use.

The project’s communication strategies also targeted religious barriers to FP use. In general, religious leaders from Catholic, Protestant or Muslim denominations in the project sites did not permit the use of FP. Nevertheless, many women went against the proscribed religious teaching and used FP anyway. Women did so to, for example, avoid unwanted pregnancy and the potentially dangerous and illegal abortion which may result.

[ … ] it is better to use family planning because to abort is like killing someone and that is condemned by law in DRC*.*

Focus group discussion, IUD users, married woman, 30-40 years old, primary education, Kasongo.

Men who went against religious teachings reported doing so mainly for the perceived economic benefits that FP could bring them.

I am Catholic and, for my Church, using these methods is a sin because you have to procreate. But this ignores the problems people face at home- it’s not the Church who has to manage our families. [ … ] It is not in my interest to follow what [the priest] says – I have to space my children*.*

Focus group discussion, partners of IUD users, married man, 50-60 years old, primary education, Kasongo

Traditional leaders tended to be in favor of FP methods as they recognized that, historically, people resorted to herbs and other natural substances as well as to spousal separation in an attempt to stop conception until the period of birth spacing was deemed appropriate. Community Health Workers built upon the perceived traditional importance of the notion of spacing to make the idea of modern methods, including the IUD (with its long duration of efficacy) more acceptable.

The findings indicate that more than the religious teachings themselves, it was communities’ interpretations of these teachings within their lived realities, that determined FP use, as long as they were aware of its potential health and economic benefits.

*Perceived advantages and disadvantages of the IUD.*

A primary perceived benefit was that the method had no or limited side-effects. In some cases, women had switched to the IUD from other methods as they were concerned about menstrual irregularities which the IUD did not seem to cause.

I started with the implant but because of its side-effects such as prolonged periods and heavy bleeding I had it taken out and replaced it with an IUD – I’m telling you, since I had the IUD inserted I’ve not had any worries*.*

Focus group discussion, IUD users, married woman, 40-50 years old, secondary education, Kasongo.

The fact that there were few perceived side-effects associated with the IUD also led some men to accept and recommend it to others. A swift return to fertility upon removal was also seen as advantageous by both women and providers. This stands in contrast to the widespread misperceptions in the region that IUD use can make sub-sequent pregnancies difficult or even render women infertile. This misperception, which had been addressed in the community messaging of the project, was not voiced by any of the participants.

In addition, the long duration of the IUD’s efficacy was perceived by both men and women to be a distinct advantage compared with other methods. They reported that this was particularly relevant in a setting where women often live far from a health facility. Increased spousal support for IUD use could be due to perceived lack of side effects and awareness of the method’s long duration appeared to increase spousal support for IUD use.

As mentioned above, however, women sometimes still needed to use a method of contraception in secret due to the disapproval of their husbands. It has been shown that discovery of women’s clandestine use can sometimes lead to divorce or gender-based violence [27]. It is therefore imperative that women who use FP clandestinely are not discovered by their partners and that they are given an opportunity to choose an appropriate method that suits their circumstances. The IUD was perceived as superior to any other method to prevent detection by unsupportive partners.

If a woman’s husband is not aware of her use, it is the best method to use as you can hide it from him over a long period of time*.*

Focus group discussion, IUD users, married woman, 30-40 years old, primary education, Kayna.

Perceived disadvantages of the method were few and were all to do with the embarrassment surrounding IUD insertion, especially with male providers.

I don’t like it because it has to be inserted through the vagina*.*

Focus group discussion, Users of other methods, married woman, 30-40 years old, primary education, Kasongo.

However, as shown in more detail below, providers were sensitized on the importance of putting women at ease in a context where the intimate nature of the insertion process can be problematic. As training records and program data showed, even though about half of trained providers were male, their clinical skills and interpersonal communication made many women feel comfortable and their gender was not a deterrent to overall increased IUD use under the project.

### Findings on supply factors related to IUD uptake

#### Supply of commodities

Project activities ensured a consistent availability of IUDs throughout the intervention area by strengthening the supply chain. This was an important pre-condition to programmatic success as the method was previously unavailable in project areas. Evidence of effective partnerships between CARE and local health services in this programmatic area was reflected in the extended coverage reported by respondents.

Yes - the IUD is available as are all the other methods because the health center places an order and they are sent as soon as it arrives at the central office*.*

In-depth interview, male community health worker, 50-60 years old, Kayna

#### Training and supervision

Within the context of a ‘rights-based approach’, provider training emphasized the technical skills needed for IUD insertion and removal. Recognizing provider bias as a potential major barrier to IUD provision, the training placed emphasis on conveying the benefits of the method including its long duration of efficacy and the swift return to fertility without compromising women’s free choice. The providers reported that they had adopted this knowledge and passed it on during FP counseling. The effectiveness of the clinical training was substantiated by a number of FP clients who remarked on the fact that they perceived the health workers who inserted their IUD to have been well trained.

I know that the provider who placed my method knows his work well. He is very well informed about family planning*.*

Focus group discussion, IUD users, married woman, 30-40 years old, secondary education, Kasongo.

Providers were regularly visited by CARE staff who provided supportive supervision. This focused on provider competence with regard to insertion and respectful interactions with clients, a crucial aspect of service quality, as evidenced by the apprehension around insertion voiced among both men and women in the focus groups. Training also emphasized the importance of routine data collection follow-up. In addition, the skills learnt by the providers were imparted more generally in the community which is likely to have an impact on IUD uptake and service quality. Project staff in the three zones worked twice a week with government health care providers in their communities training them how to counsel clients and provide FP services especially LARCs as part of their on-the-job coaching strategy.

#### Perceptions of service quality

Returning to Hancock et al’s elements of service quality [21], clients stated that they were given a choice of a full range of methods so they could freely make up their minds as to which they preferred. This was important feedback for a project that sought to balance overcoming IUD-specific barriers to FP access with the importance of women’s free and informed choice.

According to those who use the methods, they always tell us that when you go to the health center, a provider will show you all the different methods and it’s up to you to choose the one you want – s/he will tell you all the advantages and disadvantages of each one*.*

Focus group discussion, non-users, married woman, 20-30 years old, secondary education, Lubero.

Respondents perceived providers’ technical competence to be good and appreciated the fact that there was a follow-up system in the community to deal with problems, side-effects and adverse events. Women also felt reassured that they could have the IUD removed at any time without any perceived problems. The providers themselves conveyed pride in all aspects of service quality including the way they greeted their clients, recorded their details and provided outreach to remote areas. Most importantly, providers sought to guarantee client confidentiality especially given the need for secret use by some women, directly addressing one of the obstacles women faced in cases where their partners did not consent to their FP use.

When you insert the IUD you can’t tell anyone – you can’t divulge the secret. It has to remain confidential*.*

In-depth interview, male provider, 50-60 years old, Kasongo.

### Towards a theory of change to enhance IUD provision and uptake

**Fig. 2:**
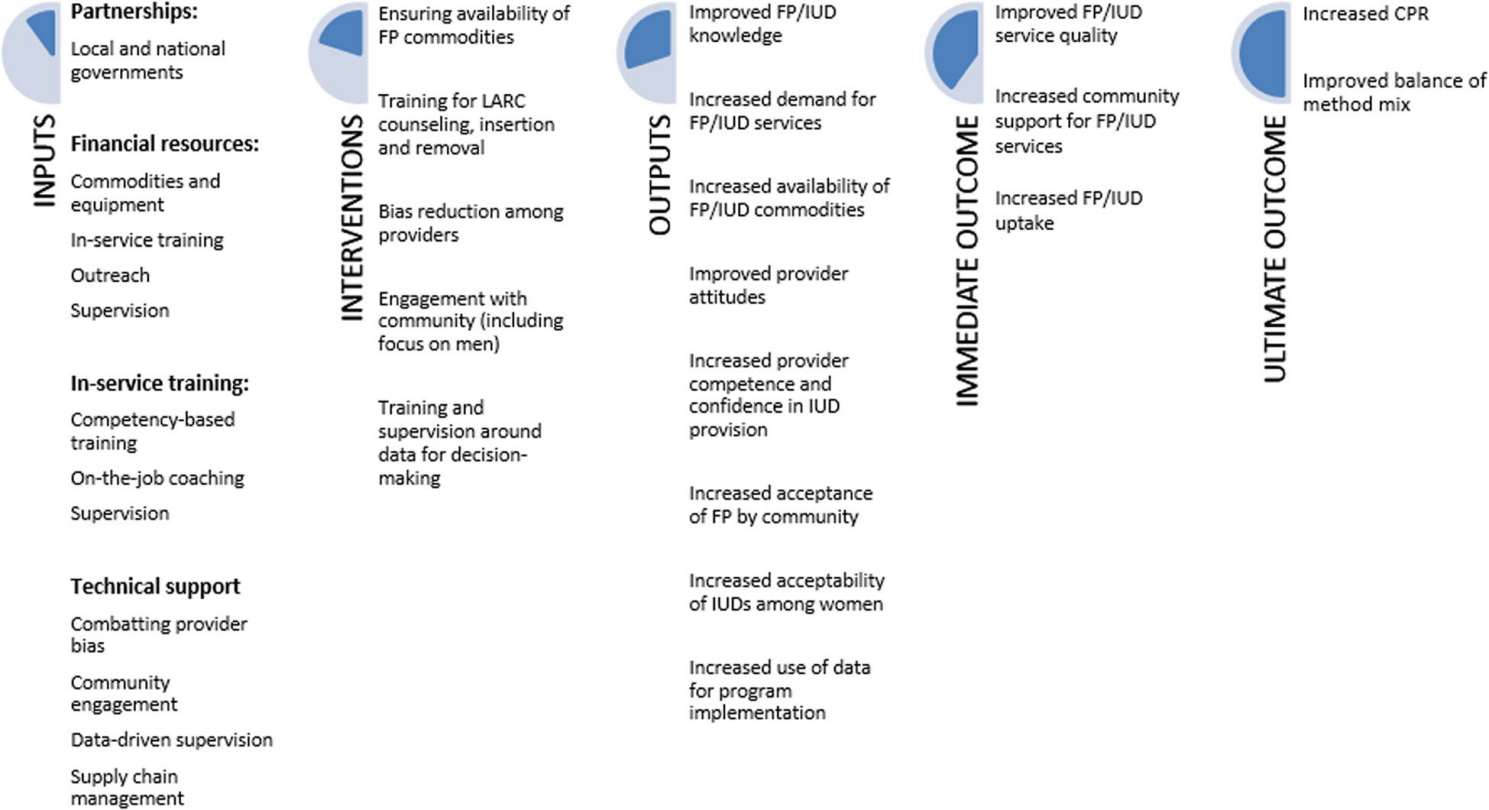
Theory of Change to enhance effectiveness of IUD access and uptake in Kasongo, Kayna and Lubero health zones in DRC

The findings presented above contributed to the development of a Theory of Change for improved IUD access and uptake (see Fig. 2). It is emphasized that the elements of the Theory of Change are context-specific and emerged from the informants’ perspectives in this Congolese setting. However, they may be replicable elsewhere although further research is needed to confirm this.

In the context of this study, rights-based access to a full range of FP methods was impeded by a lack of supplies, clinical competence and consistent supervision as well as by the presence of misconceptions and rumors surrounding FP methods among men, women and providers. In addition, the interpretation of socio-cultural and religious norms in the community further restricted women’s reproductive options. The Theory of Change presented here summarizes the inputs and interventions required to overcome these barriers, ultimately leading to increased FP uptake and a broadened method mix.

In terms of inputs, from the outset, the project required as a ‘necessary condition’ effective partnerships with local and national Government bodies. This facilitated ‘buy-in’ and ownership, so that national and local authorities were motivated to be involved in the delivery of high-quality services. An additional necessary input comprised financial resources for commodities and equipment, in-service training, outreach and supervision. Outreach and supervision were also strengthened by investment in in-service training. A key input was technical support for combatting provider bias (for example, a belief that IUDs are not appropriate for nulliparous women). In addition, the project’s success can also be attributed to ensuring that data-driven supervision and supply chain management had adequate financial and human resources. The Theory of Change also shows that the necessary interventions comprised making FP commodities available, and providing training for LARC counseling and provision. As noted above, the facilitators and community health workers carried out community mobilization which involved providing information and dispelling rumors, thus creating demand. Through the community fora they also promoted service quality and accountability. The engagement with men and community stakeholders served to strengthen the acceptability of the IUD as a viable method, even in the face of religious disapproval. Routinely collected data was analyzed in collaboration with the community and used for decision-making about program orientation. This required the training of project staff, their Government counterparts as well as community stakeholders.

Figure 2 shows that the outputs resulting from these interventions comprised increased knowledge and reduced bias about FP and about IUDs among providers, clients and the community. There was also an increased demand for FP in general but especially for IUDs (all the more remarkable given the low levels of use nationally). Availability of commodities was assured during the project’s duration by robust and effective systems of contraceptive security which minimized stockouts. The training and supervision resulted in improved provider competence and confidence with regard to IUD insertions meaning that women had a full choice of methods available. IUDs were increasingly perceived to be acceptable by women and within the community, as individual and community sensitization dispelled myths, rumors and the stigma associated with insertion.

Community sensitization about the method also led to its greater acceptability amongst men and elders, especially as messages couched the benefits of family planning within ‘world views’ which approved of traditional methods of spacing childbearing. These individual and group sensitization techniques thus create new norms which facilitated IUD uptake and, in theory, enhance sustainable provision. In addition, program planning was facilitated by the analysis of routine data and by soliciting input from community stakeholders in order to explain trends and to identify future programmatic implementation and orientation.

The resulting immediate outcomes demonstrated in the Theory of Change included improved service quality enhanced by provider training which improved skills and attitudes allowing them to meet the increased demand for LARCs. In addition, they were accountable to their communities via the fora. This is likely to lead to a sense of community support for FP and IUD services and a willingness to support the mechanisms needed to create demand and provide high quality service delivery.

Ultimately, the intervention resulted in increased FP uptake and an improved balance of method mix. By involving the community from the outset and reaching out via multiple methods of communication to women, men and stakeholders (who often act as ‘gatekeepers’ to service access), an enabling environment was created for improved IUD uptake, strengthening the likelihood of sustained outcomes. The fact that this environment is shaped by the opinions of the community itself is, in principle, likely to greatly facilitate the development of quality FP services.

## Discussion

Through this study we identified that, in the context of slow IUD uptake in sub-Saharan Africa, both supply and demand elements influenced a significant increase in IUD adoption among women in three health zones in DRC supported by a CARE project. We found this increase to be associated with programmatic inputs related to local partnerships, service quality, method availability and community engagement activities.

Further, these results show that significant unmet demand for IUDs among women in this context drive this uptake rather than provider bias. The qualitative results – contextualized by the routine monitoring data – show that expanding the method mix resulted in considerable IUD uptake, demonstrating that focused efforts on removing pre-existing barriers to IUDs represent not overexertion of provider influence but fulfillment of women’s right to a robust range of methods. This is a timely consideration given the increasing importance placed on ensuring that LARCs give adequate weight to meeting women’s unmet need for a full-range of methods and to ensuring consistent efforts to avoid provider bias against or in favor of any method [28–30].

Our study had some limitations. Purposive sampling may induce some inherent biases including a risk of homogeneity. However, the goal is not to generalize these findings to a wider population but rather to obtain insights into a phenomenon, individuals and events, in this case pertaining to the experience and perception of IUD use in this setting [31]. Only three participants in focus group discussions were single and the rest were married. Therefore unmarried, sexually active women may not have been sufficiently represented. Although translations were verified by native speakers, it was not possible to carry out back-translations. Therefore, some meanings may have been lost in the process. In addition, it is possible that desirability bias may have influenced some of the responses of the key informants, given the fact that the research was conducted by the project supporting the reproductive health services in the area. The findings from this study contributed to the development of a Theory of Change showing the inputs and interventions needed for enhanced IUD uptake.

This fills an important gap in the literature with regard to strengthening evidence on factors influencing improved use of FP in general and uptake of LARCs in particular. Existing evidence around particular program components is often limited to discussing just some of the factors presented here, or not specifically as they relate to IUDs, particularly in fragile settings such as Eastern DRC. In addition, the different levels of the change pathways behind programmatic success are rarely outlined in detail.

The use of a Theory of Change such as the one outlined in this paper can range from a broad interpretation to a narrow prescription. It can thus be applied in a variety of ways: as a technical tool for the precise application of its elements, a ‘way of thinking’ helpful in providing general direction, or as an approach for practitioners to develop a complex and nuanced understanding of how change happens, a broad interpretation they refer to as ‘political literacy’ [32]. In the view of Stein and Valters, donors, practitioners and policy makers may situate themselves differently on this continuum and this position, together with the existing resources, political will and motivations will determine how a theory of change might be used in a particular context, by what actor and for what objectives [32]. In this sense, the Theory of Change presented here may be used as a conceptual tool or a blueprint from which other programs may draw on for a specific purpose, such as strategic planning, program design, or monitoring, evaluation and learning. At the same time, consideration should be given to the sustainability of any similar programs, which could be promoted by strengthening an understanding of the importance of addressing specific supply and demand elements simultaneously and within the context of a rights-based approach and strengthened partnership. This might include a focus on continuous learning as well as specific tools such as ‘power analysis’, aimed at grounding implementation in local realities [32].

## Conclusion

The findings of the paper show that by involving local stakeholders in addressing structural and socio-cultural barriers to women’s free access to FP, programs can positively influence quality of service and method mix as well as knowledge and attitudes surrounding FP use and thus improve the uptake of FP in general and IUDs in particular, even in conflict-affected settings. A proposed Theory of Change details the causal links between the project activities the observed outcomes and may be used as guidance for developing effective FP programming elsewhere.

## Data Availability

The datasets used and/or analysed during the current study are available from the corresponding author on reasonable request.

## Abbreviations

DRC: Democratic Republic of Congo
FP: Family Planning
IUD: Intrauterine Device
LARC: Long-Acting Reversible Contraceptive
SAFPAC: Supporting Access to FP and Post-Abortion Care Project
